# Converging XGboost Machine Learning and Molecular Docking Strategies to Identify Attractants for *Ceratitis capitata*: Molecular Characterization and Database Curation of Natural Ligands for In Vitro/In Vivo Tests

**DOI:** 10.1002/arch.70095

**Published:** 2025-09-18

**Authors:** E.B. Alencar Filho, R.P. Guimarães, V.C. Santos, A.B.P. Bispo, B.A.G. Paranhos, N.C. Aquino, R. Nascimento, R.F. Oliveira Neto

**Affiliations:** ^1^ Laboratory of Molecular Modelling Applied to Pharmacy (LAMMAF)—Graduate Program in Biosciences; Graduate Program in Health and Biological Sciences Federal University of Vale do São Francisco Petrolina Pernambuco Brazil; ^2^ Department of Pharmaceutical Sciences Federal University of Vale do São Francisco Petrolina Pernambuco Brazil; ^3^ Laboratory of Biological Control Embrapa Semiárido Petrolina Pernambuco Brazil; ^4^ Chemical Ecology Laboratory, Institute of Chemistry and Biotechnology Federal University of Alagoas Maceió Alagoas Brazil; ^5^ Department of Computer Engineering Federal University of Vale do São Francisco Juazeiro Bahia Brazil

**Keywords:** *Ceratitis capitata*, ligand and structure‐based virtual screening, machine learning, odorant binding proteins, semiochemicals

## Abstract

The Mediterranean fruit fly *Ceratitis capitata* (Wiedemann) (Diptera: Tephritidae) is one of the most critical agricultural pests, causing economic damage globally due to its wide range of fruit hosts. Conventional insecticides have brought environmental, human health, and resistance challenges, driving interest in semiochemicals as sustainable pest management alternatives. Potential molecular attractants can be assessed experimentally through methods such as electroantennography (EAG) or behavioral assays. Odorant Binding Proteins (OBPs) have been recognized as crucial mediators in detecting these chemical signals. Although isolated compounds can provide mechanistic insights, volatile blends more accurately reflect natural conditions and typically elicit stronger behavioral responses. However, designing effective blends is challenging due to their complexity and regulatory limitations. Therefore, curated molecular databases of potential attractants become essential to accelerate the discovery and reduce cost in research programs, both in vitro and in vivo tests. The in silico molecular approaches, including Molecular Docking, Molecular Dynamics (MD) and Quantitative Structure–Activity Relationships (QSAR), offer cost‐effective methods to prioritize candidates and/or understand ligand‐OBP interactions. In this study, computational methodologies including Machine Learning (ML) based QSAR, molecular docking and MD simulations were integrated to highlight molecular features of standard molecules and identify potential attractors for *C. capitata*, which are expected to be good OBP binders. Initially, was applied a Bee Colony Algorithm, combined with an final XGBoost Machine Learning model, enabled the identification of five essential molecular descriptors to explain the attractant effect of 20 standard compounds recognized in the literature. Applying this model to an online database of natural products from Brazil (NuBBE—Nuclei of Bioassays, Ecophysiology and Biosynthesis of Natural Products Database), 206 molecules were identified from over 2000 candidates. In a parallel front of investigation, docking‐based virtual screening was performed using the same NuBBE database. Most promissory compounds were discussed based on binding energy, structure/geometry focusing on interactions and estimated volatility, through the evaluation of vapor pressure. MD simulations with the gold standard compound (E,E)‐α‐farnesene provided insights into ligand‐protein interactions. Interestingly, 16 of the top 20 ranked compounds after dockings were predicted as attractors by the XGBoost model. Finally, the curated database of 206 compounds, the great contribution of this paper (beyond the model), can be used to assertively select molecules for experimental tests of future blends or isolated compounds.

## Summary

- Bee Colony + Best First Search algorithms demonstrated high variable filtering power, followed by a XGBoost QSAR model with excellent validation parameters for predicting natural attractants of *Ceratitis capitata* medfly.
- Structure‐based virtual screening (docking and molecular dynamics) in the NuBBE natural products database identified potentially promising compounds and had good correspondence with the QSAR results.
- The application of the Machine Learning‐based QSAR model to the NuBBE natural products database (> 2000 compounds) allowed the construction of a database of 365 molecules classified as potential attractants, 206 of which had a higher probability (> 0.7), for in vitro and in vivo tests.

## Graphical abstract

By integrating XGBoost‐based QSAR, molecular docking, and dynamics, we identified 206 natural compounds from Brazilian biodiversity as promising attractants for *Ceratitis capitata*. This curated library provides a cost‐effective resource to guide in vitro and in vivo testing, advancing sustainable semiochemical‐based pest management strategies.

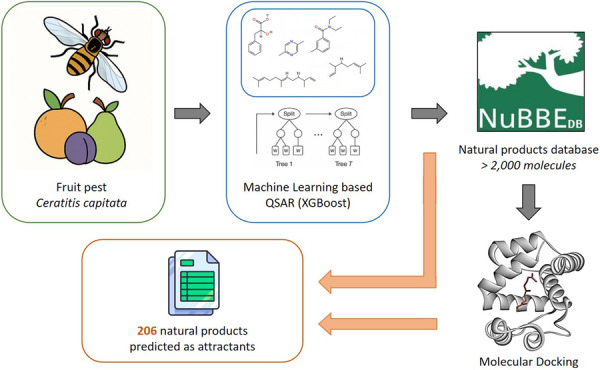

## 1 Introduction

The Mediterranean fruit fly, *Ceratitis capitata* (Wiedemann) (Diptera: Tephritidae), is globally recognized as one of the most concerning agricultural pests (Liquido et al. 2017; Malacrida et al. 2007). Its remarkable ecological adaptability and polyphagous capacity, attacking approximately 400 fruit varieties, classify it as an invasive pest. The insect impacts significantly the fruit production, both through direct damage and facilitating preharvest infections. In Brazil, particularly in the Northeast and Southeast regions with intense fruit cultivation, *C. capitata* represents one of the economically most relevant pests, complicating exports and requiring quarantine measures (Haji et al. 1991; Araujo et al. 2022).

Conventional pest control in Brazil is predominantly chemical, relying on synthetic insecticides such as organophosphates and pyrethroids. However, indiscriminate pesticide use raises environmental concerns, human health issues, and insect resistance (Isman 2020). As an alternative, biological control of *Ceratitis capitata* has focused mainly on the use of parasitoids and sterile insect technique (SIT). Parasitoids such as *Diachasmimorpha longicaudata* have been successfully released in several regions, reducing fruit fly populations through natural parasitism (Montoya et al. 2000). The SIT, based on the mass release of sterile males, has also been applied in integrated programs worldwide, showing strong effectiveness when combined with other measures (Enkerlin et al. 2015). More modern strategies include the use of genetic technologies, such as transgenic strains with conditional lethality or CRISPR‐based gene drives to suppress pest populations (Alphey 2014; Raban et al. 2023). In addition, semiochemical‐based strategies (pheromone dispensers as well as attract‐and‐kill formulations) represent environmentally sustainable tools complementing classical control. These advances illustrate a shift toward precision, environmentally friendly, and target‐specific approaches in fruit fly management. The semiochemicals are signaling compounds produced and released in minimal amounts, modulating essential insect behaviors such as food identification, oviposition site selection, and partner location (Witzgall et al. 2010). Manipulating chemical communication through the identification of new attractants (or repellents) can offer a promising and environmentally sustainable approach for fruit fly population control.

Olfactory sensitivity is crucial for insect survival and reproduction. Odorants, including pheromones (produced by insects for intersexual communication, such as the (E,E)‐α‐farnesene released by male *C. capitata*), and host plant volatiles, are detected by antennal olfactory sensilla. Odor perception involves Odorant Binding Proteins (OBPs), small extracellular proteins highly abundant in the sensillar lymph of antennae (Pelosi et al. 2014). OBPs bind hydrophobic odorants, transporting them to Olfactory Receptors (ORs) located in the dendritic membranes of olfactory sensory neurons, converting olfactory signals into behavioral responses. OBPs also act as the initial molecular filter for semiochemical discrimination. The 3D structure of OBP from *Ceratitis capitata* (PDB ID: 6HHE) is available and is known for its high expression in females and its role in intersexual olfactory communication (Siciliano et al. 2014). Structurally, insect OBPs contain a domain of six α‐helices forming a hydrophobic cavity stabilized by disulfide bridges, while *C. capitata* OBP has an additional seventh C‐terminal α‐helix enhancing its binding cavity (Falchetto et al. 2019).

Although testing isolated compounds can offer mechanistic insights, the behavioral responses of *C. capitata* are often stronger when exposed to volatile blends, which more accurately reflect natural odor profiles (Ng et al. 2021). These blends tend to act synergistically, but their formulation and validation remain complex due to the diversity of natural emissions and regulatory constraints. Therefore, the availability of curated databases containing molecules with known or predicted olfactory activity is essential to accelerate the discovery of new attractants. In this context, in silico strategies such as molecular docking, virtual screening, and Quantitative Structure–Activity Relationship (QSAR) modeling are powerful tools to prioritize candidate ligands, investigate their interactions with OBPs, and support the rational design of behavioral assays (Ouabane et al. 2023; Ouabane, Zaki, Alaqarbeh et al. 2024; Naanaai et al. 2025; Zaki et al. 2024).

Machine Learning (ML) algorithms can be integrated to model and predict the relationship between molecular descriptors and biological activity, guiding compound selection more efficiently (Wu et al. 2021; Oliveira Neto et al. 2025; Ouabane, Zaki, Tabti et al. 2024), characterizing a QSAR. Feature selection plays a critical role in QSAR modeling, by reducing dimensionality and improving model generalization. Among modern heuristic strategies, the Artificial Bee Colony (ABC) algorithm has gained attention due to its effectiveness in exploring large and complex search spaces, mimicking the intelligent foraging behavior of honeybee swarms (Lin et al. 2021). When combined with Best‐First Search (BFS), a greedy algorithm that prioritizes descriptor subsets with optimal evaluation scores, this hybrid method enables the identification of a reduced yet highly informative set of molecular descriptors.

For model training, eXtreme Gradient Boosting (XGBoost) has emerged as a powerful ML algorithm for classification tasks in cheminformatics. It is based on an ensemble of decision trees trained sequentially to minimize classification error, offering high performance, scalability, and resilience to overfitting (Chen and Guestrin 2016). The integration of ABC‐BFS feature selection and XGBoost modeling reflects the state‐of‐the‐art in QSAR workflows, enhancing both interpretability and predictive accuracy in virtual screening studies.

In another perspective, Structure‐Based Virtual Screening (SBVS) enables selecting compounds from large databases with high interaction potential for target proteins. Molecular docking, central to SBVS, predicts the preferred conformation and orientation of a ligand in the binding site of a macromolecule, calculating their binding energies (Meng et al. 2011; Zrinej et al. 2025). To validate and refine docking outcomes, Molecular Dynamics (MD) simulations evaluate the temporal profiles of ligand‐target complexes, accounting for their flexibility and aqueous environment interactions, providing more accurate analysis (Hollingsworth and Dror 2018; Naanaai et al. 2024).

This study aimed to build a database of Brazilian natural products predicted as most likely attractors for *Ceratitis capitata*, also making notes on chemical affinity in OBP and structure–activity relationships. A Bee Colony Optimization algorithm, combined with XGBoost machine learning model, was applied to a data set of standard attractants and related compounds, resulting in a QSAR model that identified five important molecular descriptors to discriminate attractant and non‐attractant small compounds. This model was used to determine 206 promising natural compounds from an initial pool of over 2000 from NuBBE online database, being suitable for experimental validation either individually or as components of attractant blends. Additionally, virtual screening via molecular docking was performed in the same NuBBE database to verify the compounds with the greatest affinity for the OBP of *Ceratitis capitata*, comparing the structure‐based results with the ligand‐based ones. Specific objectives included identification of promising compounds, analysis of intermolecular interactions, and perform molecular dynamics simulations to refine inferences with the standard attractant ((E,E)‐α‐farnesene).

The machine learning method proved to be powerful in discriminating attractor compounds and, together with the generated database (206 compounds), can be used in the arduous task of identifying potential compounds and understanding the semiochemical mechanism for *Ceratitis capitata*.

## 2 Computational Procedures

### 2.1 Ligand‐Based Approaches

#### 2.1.1 Training Data Set Selection

A total of 45 compounds were used for training Machine Learning model (Table 1). Among them, 29 molecules were retrieved from an specific paper (Tabanca et al. 2019), comprising aromatic compounds previously reported in olfactory or behavioral assays against *Ceratitis capitata*. An additional 16 structurally similar or functionally relevant compounds were selected through a comprehensive literature search in databases such as PubChem (Kim et al. 2023), SciFinder, and Scopus, focusing on volatiles with reported insect semiochemical activity (Siciliano et al. 2014; Falchetto et al. 2019; Tabanca et al. 2019; Tan et al. 2014; Ohinata et al. 1979; Jacobson et al. 1973; Baker et al. 1990; Merli et al. 2018). In the paper by Tabanca et al. (2019), only four compounds were considered attractants. Together, 20 active molecules and 25 inactive molecules were used for training. The first set was assigned with the binary classifier 1 (attractant) and the second 0 (non‐attractant).

**Table 1:** True attractant ligands considered as standard compounds.

	True ligands	2D structure	Source (doi)
1	Phenyllactic acid	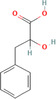	10.3390/molecules24132409
2	Estragole	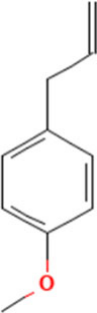	10.3390/molecules24132409
3	*o*‐Eugenol	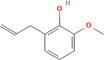	10.3390/molecules24132409
4	2‐Allylphenol	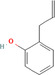	10.3390/molecules24132409
5	(E,E)‐α‐Farnesene	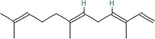	10.1016/j.ibmb.2014.02.005
6	Trimedlure	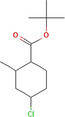	10.3390/molecules24132409
7	Ceralulre B1	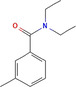	10.1007/978‐94‐017‐9193‐9_2
8	Ethyl (E)‐oct‐3‐enoate	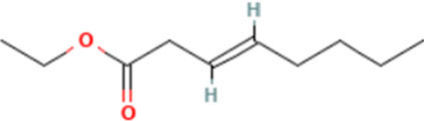	10.1007/978‐94‐017‐9193‐9_2
9	(Z)‐Beta‐ocimene	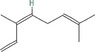	10.1016/j.ibmb.2014.02.005
10	Geranyl acetate	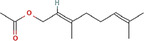	10.1016/j.ibmb.2014.02.005
11	Methyl (E)‐6‐nonenoate	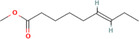	10.1093/jee/72.4.648
12	(E)‐6‐Nonen‐1‐ol	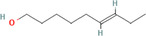	10.1021/jm00261a018
13	2,3‐Dimethylpyrazine (2,3 DMP)	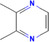	10.1093/jee/83.6.2235
14	2,5‐Dimethylpyrazine (2,5 DMP)	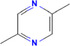	10.1093/jee/83.6.2235
15	(E)‐β‐Farnesene	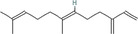	10.1111/imb.12559
16	3‐Methylbutanol	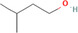	10.1111/imb.12559
17	2,3‐Butanediol	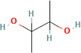	10.1111/imb.12559
18	(E)‐β‐Myrcene	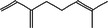	10.1111/imb.12559
19	(E)‐Beta‐ocimene	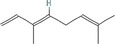	10.1016/j.ibmb.2014.02.005
20	2‐Ethylhexanoic acid	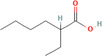	10.1007/s10886‐018‐0939‐z

#### 2.1.2 Molecular Descriptors Calculations

All the 45 compounds were converted to three‐dimensional molecular structures using ChemSketch software (ACD/Labs 2022). Geometry optimizations were performed at PM3 semiempirical quantum level (Stewart 1989). The resulting structures were submitted to Dragon 7.0 software (Talete 2025) for the calculation of molecular descriptors. Only descriptors that were defined for all the compounds and exhibited any variance were retained. Then, a total of 2217 descriptors were initially obtained, covering topological, geometrical, electronic, and hybrid categories.

#### 2.1.3 Features Selection

To reduce dimensionality and select the most informative variables, the Bee Colony Optimization (BCO) algorithm (Random Forest based) was employed at Google Colab environment (Google 2025), using Python 3.10, scikit‐learn and xgboost libraries. A k‐fold cross validation was made here using *k* = 5. The BCO was used in combination with a Best‐First Search (BFS) wrapper strategy to select subsets of descriptors with highest relevance for classification. This approach was recently applied by Lin et al. (2021), who demonstrated that a Artificial Bee Colony (ABC) algorithm, combined with greedy wrapper strategies, improves descriptor selection in QSAR modeling.

All the python based codes applied at Google Colab environment (Google 2025), used in this and in the next topics of the Machine Learning analysis, are available at GitHub (https://github.com/rosalvoneto/supporting-material-insect-biochem-2025).

#### 2.1.4 Model Training and Validation

A selected subset of five molecular descriptors was used to train a binary classification model using the XGBoost algorithm (Chen and Guestrin 2016). Model development included fivefold cross‐validation and hyperparameters: n_estimators = 10; max_depth = 2; learning_rate = 0.1; subsample = 0.8; colsample_bytree = 1.0; reg_alpha = 0.1; reg_lambda = 1.0; use_label_encoder = False; eval_metric = “logloss”; random_state = 42. Internal validation metrics included: Accuracy, Sensitivity, Specificity and the Area Under the ROC Curve (AUC). Reinforcing this information, the model was built and tested in a Google Colab environment, and all scripts were written in Python using pandas, numpy, xgboost, sklearn, and matplotlib.

#### 2.1.5 Prediction and Output Evaluation

The trained model was used to predict the biological class (attractant/non‐attractant) of the molecules downloaded from NuBBE (Pilon et al. 2017) database, in the.mol format. NuBBE (Nucleus of Bioassays, Biosynthesis and Ecophysiology of Natural Products) is a research group that maintains an online database containing > 2200 molecules isolated from Brazilian plants, with a focus on the bioprospecting of compounds with biological potential. The five molecular descriptors were obtained by Dragon 7, the classifications as well as the resulting probabilities were interpreted as the model's confidence in each classification.

The use of a natural product database for ligand and structure‐based virtual screening for *Ceratitis capitata* is grounded in both evolutionary rationale and practical advantages for sustainable pest management. Natural compounds, particularly those derived from plants, represent chemically diverse scaffolds that have co‐evolved with biological systems over millions of years. This coevolution has led to structural compatibility between plant volatiles and insect olfactory receptors, making natural products highly relevant candidates for modulating insect behavior (Wink 2003; Bakkali et al. 2008).

Moreover, natural products offer advantages in terms of environmental safety and biodegradability, aligning with the goals of integrated pest management (IPM) and sustainable agriculture (Isman 2020). Unlike many synthetic insecticides, plant‐derived compounds often exhibit lower toxicity to nontarget organisms and reduced persistence in ecosystems. Using a curated virtual library such as NuBBE, which contains structurally diverse molecules isolated from Brazilian flora, enables a rational, targeted, and eco‐conscious approach to developing new attractants for *C. capitata*.

### 2.2 Structure‐Based Approaches

#### 2.2.1 Target Protein Preparation

The three‐dimensional structure of the *Ceratitis capitata* Odorant Binding Protein (OBP), PDB code 6HHE, was obtained from the Protein Data Bank (RCSB PDB 2024; Berman 2000). This macromolecule was chosen because it is the only one from the fruit fly (*Ceratitis capitata*) with a crystallographic structure available in the PDB, ensuring greater reliability for docking and ligand design, in the initial perspective of this paper. Protein preparation involved the removal of water molecules using Chimera software (Pettersen et al. 2004) and the calculation of electrostatic analysis with pH adjustment to 6.5, considered optimal for macromolecule interaction, via the ABPS virtual platform (Jurrus et al. 2018). The grid box dimensions for docking were defined using the graphical interface of AutoDock Tools (Morris et al. 2009), with coordinates X = 18.082Å, Y = 3.943Å, and Z = −7.219Å, and a box size of 20 × 20 × 20Å, with the standard Vina spacing of 1.0Å, at light of the definitions for the binding site of OBP present in the work of Falchetto et al. (2019). Although the OBP 6HHE did not contain co‐crystallized ligands for redocking, (E,E)‐α‐farnesene was selected as a reference molecule for comparisons of binding energy and most important amino acid residues in the recognition, based on evidence from the literature (Falchetto et al. 2019; Guimarães et al. 2025).

#### 2.2.2 Molecular Docking Virtual Screening and Validation

Virtual screening was performed through molecular docking using compounds from the NuBBE database (Pilon et al. 2017). The software AutoDock Vina (Eberhardt et al. 2021; Trott and Olson 2010) was used for all docking procedures. All molecules were downloaded in “.ZIP” format, extracted, and converted to “.pdbqt” format using the PyRx software (Dallakyan and Olson 2015).

In the case of *C. capitata*, which relies heavily on olfactory cues for host localization and mating, plant‐derived volatiles are key components in chemical communication (Light et al. 1988; Ng et al. 2021). Odorant Binding Proteins (OBPs) play a crucial role in this process, acting as the first molecular interface between environmental odorants and olfactory sensory neurons. Therefore, screening compounds from natural sources (especially those originating from native or ecologically relevant flora) enhances the chances of identifying ligands that effectively interact with OBPs and influence behavior in a biologically meaningful way (Pelosi et al. 2018; Ouabane, Alaqarbeh, Hajji et al. 2024).

After docking virtual screening, the 20 molecules with the highest binding affinities were highlighted. From this group, five molecules were selected as best solutions, prioritizing those with the most negative binding energy and vapor pressure parameters, indicative of volatility. In the study of semiochemicals, evaluating the volatility of a compound, alongside its binding affinity to the target protein, is essential, as both properties influence its ability to reach and activate olfactory receptors under natural conditions. Enthalpy of vaporization and boiling point values were obtained from the ChemSpider platform (Pence and Williams 2010), and vapor pressure was calculated using the Clausius–Clapeyron equation through the online Omni Calculator—Vapor Pressure Calculator.

The possible interactions between the selected ligands and the OBP were visualized using BIOVIA Discovery Studio software (BIOVIA 2015), which generates 2D diagrams of the bonds between ligand atoms and protein amino acid residues, as well as three‐dimensional representations of the complex.

At this stage, it is important to clarify that the interaction mechanism between pheromones and insect olfactive structures is both subtle and diffuse. Although farnesene is considered a reference for affinity, theoretical calculations tend to overestimate interaction energies due to the absence of polar functional groups. In other words, while apolar interactions are relevant in this context, they often result in moderate to low docking scores. This should be kept in mind when interpreting the results. In this case, we believe that the geometries and interacting amino acid residues may provide more meaningful insights than the binding energy values alone. In the semiochemical context, we consider that ligand‐centered approaches, such as QSAR, are more likely to yield successful predictions in future studies than docking‐dynamics outcomes. Therefore, these structure‐based methods should be used from complimentary viewpoint.

#### 2.2.3 Molecular Dynamics Simulations

Molecular dynamics simulation procedures were carried out for the reference molecule, (E,E)‐α‐farnesene, aiming to refine the molecular docking observations and support future inferences for other compounds. Calculations were performed using GROMACS version 5.1.2 (Hess et al. 2008; Abraham et al. 2015) with the GROMOS 54a7 force field (Schmid et al. 2011). Topological parameters for the ligands were obtained via the Automated Topology Builder (ATB) online platform (Stroet et al. 2018). Protein–ligand complexes were solvated in a cubic box filled with SPC (Simple Point Charge) water molecules under periodic boundary conditions (Berendsen et al. 1984) to simulate a realistic aqueous biological environment.

The ligand choice serves as a well‐established reference, and MD was prioritized for farnesene to gain deeper insights into its interaction dynamics with the OBP, considering the computational cost of MD and the exploratory nature of the screening.

Long‐range electrostatic interactions were handled using the Particle Mesh Ewald algorithm (Darden et al. 1993), ensuring system charge neutrality. Bond lengths were constrained using the P‐LINCS algorithm (Hess et al. 1997). The system underwent energy minimization, followed by NVT (constant temperature, constant volume) and NPT (constant temperature, constant pressure) equilibration phases at 300 K, with a time step (dt) of 2 fs over 100 ps, using a modified Berendsen thermostat and a Parrinello–Rahman barostat (Parrinello and Rahman 1981). The production phase of the MD simulation was conducted for 100 ns at 300 K and 1 bar pressure, with a 2 fs integration time and data collection every 10 ps. Simulations were executed on the CENAPAD/UFC computing cluster.

The average binding free energy (ΔG) of the complexes was estimated using the MM/PBSA method (Molecular Mechanics/Poisson–Boltzmann Surface Area), analyzing frames collected from 90 to 100 ns at 10 ps intervals (1 000 frames). Calculations were performed using the “g_mmpbsa” executable (Kumari et al. 2014), a tool compatible with GROMACS.

### 2.3 Use of Artificial Intelligence Tools

Some parts of the manuscript in Introduction and Discussion sections were written with the assistance of ChatGPT (OpenAI, San Francisco, CA, USA, GPT‐4, May 2025), to improve clarity and grammar. Similarly, some scripts for algorithms were written with the assistance of ChatGPT. All content was reviewed and revised by the authors to ensure accuracy and originality.

## 3 Results

### 3.1 Machine Learning Based QSAR

The application of the ABC algorithm combined with Best‐First Search (BFS) as a wrapper method resulted in an optimal subset of five molecular descriptors from the initial pool of over 2,000 variables calculated using Dragon 7.0. This hybrid feature selection approach significantly reduced dimensionality while retaining the most relevant features for classification of active compounds against *Ceratitis capitata*. The selected descriptors were Mor27v (a 3D‐MoRSE weighted by van der Waals volume), H1s (atom‐centered fragment descriptor representing hydrogen at position 1), Eig09_AEA(bo) (eigenvalue of the autocorrelation matrix weighted by bond order), P_VSA_e_1 (sum of van der Waals surface areas with low electronegativity), and R6p+ (GETAWAY descriptor reflecting the influence of atomic polarizability in topological space). Each descriptor captures distinct aspects of molecular topology, geometry, and electronic behavior, which can also explain the interactions with odorant‐binding proteins (OBPs) and olfactory receptors:

**Mor27v** (a 3D‐MoRSE descriptor weighted by van der Waals volume) encodes information about the spatial distribution of atomic van der Waals interactions within a defined distance class (27Å). These descriptors, developed from electron diffraction functions, encapsulate molecular geometry and bulkiness, which are crucial for optimal accommodation within the hydrophobic cavity of an OBP.

**H1s** descriptor (H autocorrelation of lag 1, weighted by atomic I‐state) is a classical parameter from the GETAWAY family, which integrates both topological and electronic information of a molecule. It evaluates the autocorrelation between atoms directly connected by a covalent bond (lag 1), weighting this relationship by the I‐state, a measure of the intrinsic electronic capacity of each atom based on its valence and connectivity. In this way, H1s reflects the degree of electronic similarity or contrast between neighboring atoms, being higher in homogeneous structures such as simple hydrocarbons, and lower in molecules with heteroatoms or polarized bonds, where differences in I‐state are more pronounced. This descriptor thus translates how local electronic distribution is organized along covalent bonds, capturing subtle structural aspects that influence physicochemical and biological properties, such as polarity, solubility, and reactivity patterns.

**Eig09_AEA(bo)** captures the ninth eigenvalue of the bond‐order‐weighted adjacency matrix (electronic autocorrelation). This topological descriptor integrates both bond order and electronic connectivity, reflecting molecular rigidity and extent of conjugation. Structures with higher resonance or conjugated systems often display enhanced shape complementarity with protein binding sites.

**P_VSA_e_1** is part of the van der Waals surface area (VSA‐based) class, summing areas of atoms whose partial charge lies within a specified range (e.g., low electronegativity). This descriptor is associated with lipophilicity and surface exposure. Elevated P_VSA_e_1 values suggest significant nonpolar surface area, which favors interaction via hydrophobic forces within OBP cavities.

**R6p+** is a GETAWAY descriptor that assesses spatial distribution of atomic polarizability within a molecular framework. It captures how polarizable atoms are topologically arranged over six‐bond paths. Enhanced polarizability may facilitate induced‐fit interactions with the OBP, improving binding dynamics and specificity.

Together, these descriptors suggest that attractive activity may be influenced by a combination of molecular size, shape, surface lipophilicity, polarizability, and electronic distribution, all of which are relevant for molecular recognition by olfactory proteins. These findings are consistent with prior studies highlighting the importance of hydrophobic interactions and molecular volume in the binding of volatile semiochemicals to insect OBPs (Leal 2013; Pelosi et al. 2018).

The final predictive QSAR model was built using the XGBoost algorithm trained on the selected subset of five molecular descriptors (Mor27v, H1s, Eig09_AEA(bo), P_VSA_e_1, R6p+). The model was validated internally using fivefold cross‐validation, yielding a mean AUC of 0.963, with individual fold AUCs consistent with good and balanced generalizations (Table 2). After validation, the model was applied to a data set of natural products from the NuBBE database. Out of over 2100 molecules evaluated, 365 molecules were classified as attractants (class 1), where 206 compounds were predicted with higher confidence (probability > 0.7). A probability value > 0.7 in XGBoost classification indicates that the model is more confident that the sample belongs to the positive class. We hope that this database will be used to save costs and time for experimental research groups, to use the most convenient compounds indicated by us for individual tests or the composition of blends. A full list of the predicted attractants, along with their SMILES representations, IUPAC or PubChem common names (when available), predicted effect (attractant = 1) and probabilities is available in .xlsx Supporting Information S1.

**Table 2:** Validation parameters for XGBoost ML modelling.

	Fold					
	1	2	3	4	5	Mean
AUC	0.775	0.950	0.950	0.950	0.875	0.963

**Figure 1:**
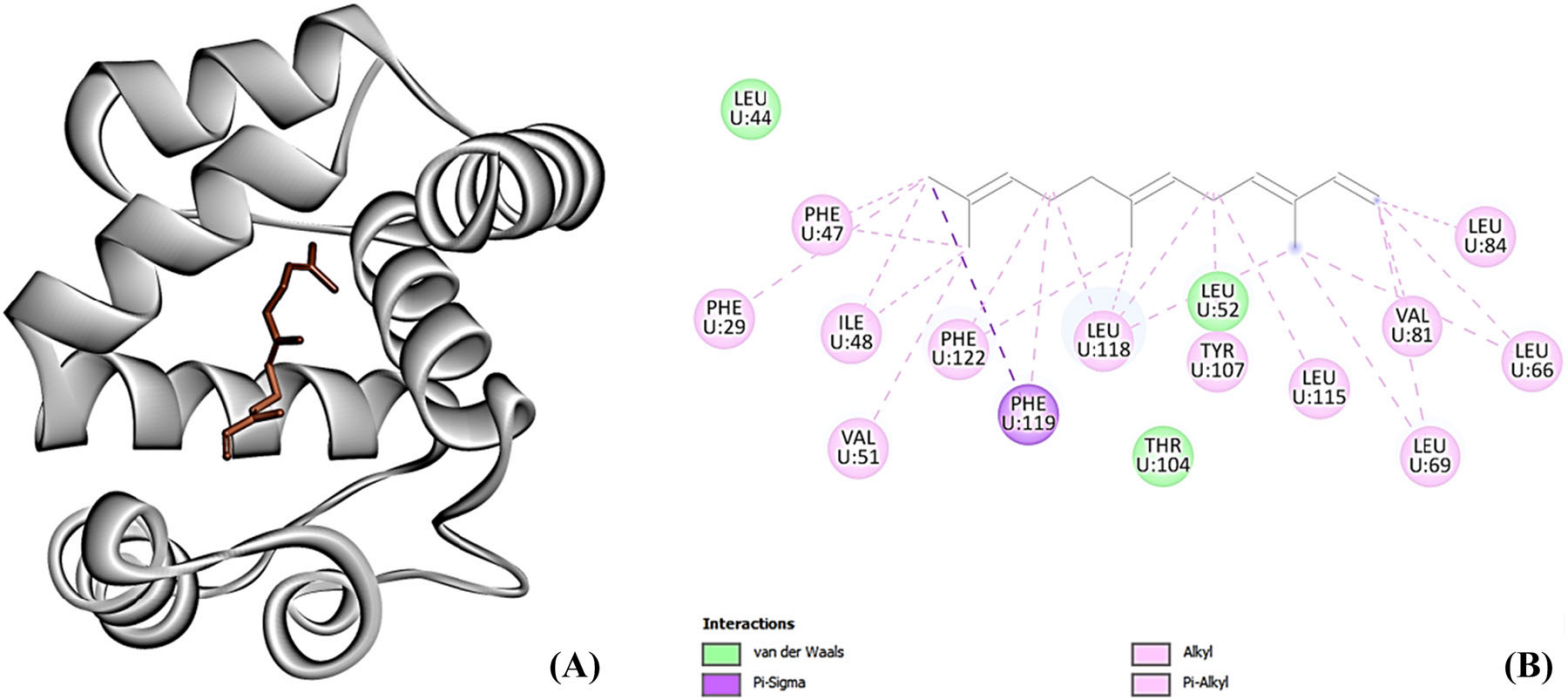
(A) 3D structure of farnesene binded to OBP from *Ceratitis capitata*; (B) 2D interactions map.

The target macromolecule for virtual screening was the Odorant Binding Protein (OBP) of *Ceratitis capitata*, identified as 6HHE in the Protein Data Bank, with a resolution of 1.52Å (Figure 1). The reference compound selected for comparison, (E,E)‐α‐farnesene, exhibited a binding energy of –7.9 kcal/mol with the OBP. The interactions observed included pi‐sigma, alkyl, pi‐alkyl, and van der Waals forces, primarily involving nonpolar and aromatic regions of amino acid residues such as PHE29, PHE47, PHE119, PHE122, ILE48, THR104, VAL51, VAL81, TYR107, LEU115, LEU44, LEU52, LEU84, LEU69, LEU66, and LEU118 (Figure 1).

**Figure 2:**
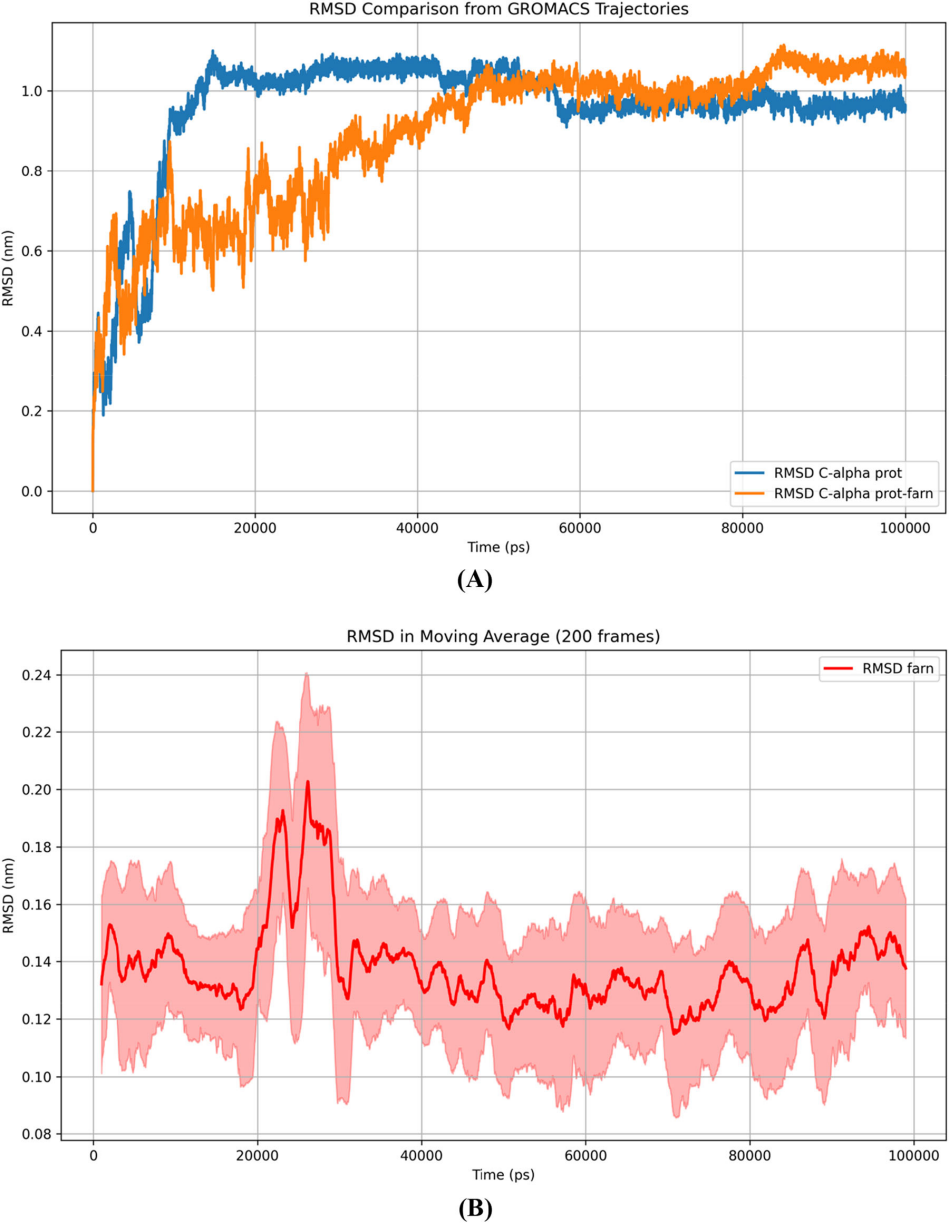
Molecular dynamics simulation plots. (A) RMSD for free protein (blue) and bound to farnesene (orange); (B) RMSD plot for farnesene as the moving average at 200 frames for better visualization (standard deviation in shaded).

Focusing on the Molecular Dynamics (MD) results for (E,E)‐α‐farnesene, the parameters evaluated were the protein RMSD (Root Mean Square Deviation) (Figure 2A); ligand RMSD (Figure 2B) as well as the binding energy calculated by MM/PBSA.

In Figure 2A, the blue line represents the evolution of the unbound odorant‐binding protein geometry. After approximately 15,000 ps, the protein reaches an equilibrium state with an RMSD value stabilizing around 0.1 nm (1Å). The orange line shows the protein complexed with (E,E)‐α‐farnesene, indicating that the protein backbone remains closer to the original complexed geometry for some time, only later reaching an equilibrium conformation (after 50,000 ps). The presence of a ligand can change the stabilization profile of a small protein, as is the case here. Considering that a static crystallographic geometry was used in the bound and unbound cases, it is plausible to observe a longer delay until the bound protein stabilizes. The most important aspect of dynamics is whether or not the system reaches a satisfactory equilibrium after a certain period of time (a range of no more than 0.2 nm of fluctuation).

The RMSD of the ligand (Figure 2B) shows that farnesene remained relatively stable during the simulation, with a variation of less than 0.2 nm along the trajectory and with reaching less than 0.2 nm (2Å) from the source geometry (from docking). This indicates that the representation provided by the docking is maintained with the relaxation of the system, and its inferences of interacting amino acids made above are relevant.

The calculated binding free energy obtained through the MM/PBSA approach was −134.722 kJ/mol, indicating a highly stable and favorable interaction between the ligand and the receptor. The value falls within the expected range for stable ligand–receptor complexes, as reported in benchmarking studies (Hou et al. 2011). In molecular dynamics simulations, binding energies lower than −100 kJ/mol are generally considered to represent strong and specific interactions. The significantly negative value observed here suggests that the ligand remains tightly bound within the binding site throughout the trajectory, reflecting a thermodynamically stable complex likely to persist under physiological conditions.

The predominantly lipophilic environment of the OBP binding site may explain Table 3, favoring the accommodation of molecules as farnesene. The overall binding was dominated by Van der Waals contributions (−139.605 ± 6.890 kJ/mol), consistent with the hydrophobic and nonpolar nature of the ligand, a sesquiterpene hydrocarbon. Electrostatic interactions contributed minimally (−1.648 ± 0.922 kJ/mol), as expected. Although the polar solvation term was positive (+23.374 ± 3.905 kJ/mol) (representing the energetic cost of desolvating polar groups) the strong nonpolar solvation contribution (SASA, −16.843 ± 0.893 kJ/mol) helped offset this effect. These findings suggest, in fact, that the binding is primarily driven by dispersion forces and hydrophobic effects, leading to a stable complex formation.

**Table 3:** Terms of energy from MD calculations for farnesene–OBP complex.

Van der Waals	Electrostatic	Polar solvation	SASA	Binding energy
−139.605	−1.648	23.374	−16.843	−134.722
+/−6.890	+/−0.922	+/−3.905	+/−0.893	+/−6.867

**Figure 3:**
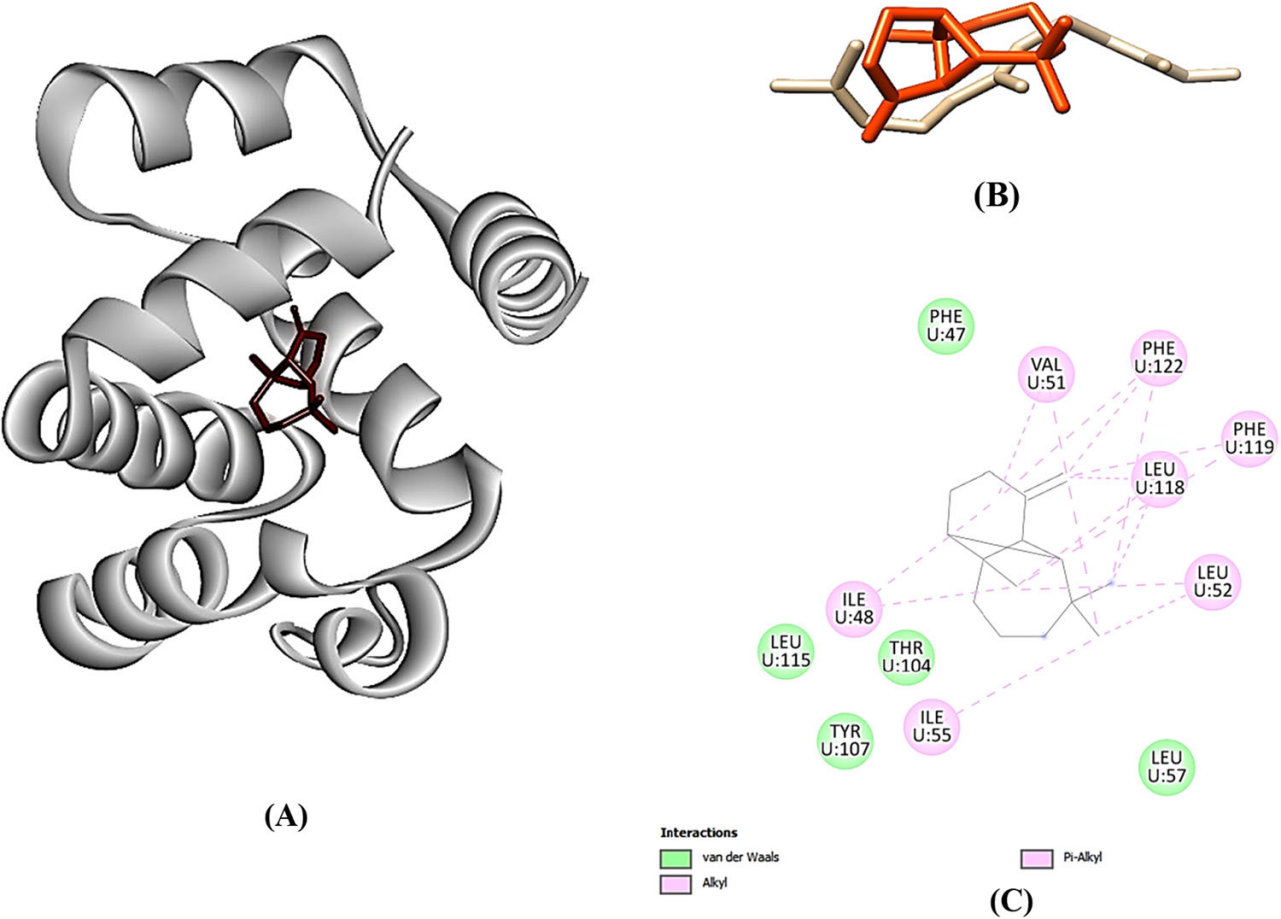
(A) 3D binding representation of β‐longipinene in OBP; (B) superimposition of standard farnesene and β‐longipinene (orange); (C) 2D interactions map for β‐longipinene.

The virtual screenings via molecular docking using the NuBBE database identified several molecules with higher binding affinities than the reference compound ((E,E)‐α‐farnesene, being the five first ranging from –9.2 to –8.5 kcal/mol (Table 4). Most of these compounds belong to the sesquiterpene class. The top five molecules were β‐longipinene (–9.2 kcal/mol), α‐copaene (–8.8 kcal/mol), α‐humulene (–8.6 kcal/mol), β‐chamigrene (–8.6 kcal/mol), and α‐selinene (–8.5 kcal/mol) (Table 3). β‐longipinene (28.684 Pa) and α‐copaene (32.2 Pa) were the most volatile; α‐humulene (11.155 Pa), β‐chamigrene (12.53 Pa), and α‐selinene (14.104 Pa) exhibited lower vapor pressures, closer to farnesene. The molecular interactions were predominantly alkyl and pi‐alkyl, with additional contributions from pi‐sigma and Van der Waals forces, involving mainly the amino acid residues VAL51, PHE119, PHE122, LEU52, LEU118, ILE48, ILE55, LEU57, LEU115, PHE47, THR104, and TYR107, illustrated for β‐longipinene (Figure 3).

**Table 4:** Docking binding energies and physico‐chemical parameters for the 20 best scored compounds after virtual screening (n.f. = not found).

Entry	Name	XGBoost class	Binding energy (kcal/mol)	Vapor enthalpy (kJ/mol)	Boiling point (°C)	Vapor pressure (Pa)
1	(E,E)‐α‐Farnesene	1	−7.9	49.8	279.6	9.708
NuBBE_1945	β‐Longipinene	1	−9.2	46.9	251.6	28.684
NuBBE_940	α‐Copaene	1	−8.8	46.6	248.5	32.2
NuBBE_1735	α‐Humulene	1	−8.6	49.4	276.3	11.155
NuBBE_1936	β‐Chamigrene	1	−8.6	49.1	273.2	12.53
NuBBE_1927	α‐Selinene	1	−8.5	48.8	270.0	14.104
NuBBE_2289	Thujopsene	1	−8.4	47.4	256.5	23.777
NuBBE_2290	(−)‐Cyperene	1	−8.4	48.7	268.9	14.68
NuBBE_1732	Alpha‐*cis*‐bergamotene	1	−8.3	n.f.	—	—
NuBBE_2294	Guaiazulene	0	−8.2	52.4	305.4	3.602
NuBBE_1350	Guaiol	1	−8.1	68.3	288.0	0.24985
NuBBE_1499	Isocaryophyllene	1	−8.1	48.6	268.4	15.1
NuBBE_1933	(−)‐α‐Himachalene	1	−8.1	48.6	268.4	15.1
NuBBE_2268^a^	1,4‐Dimethyl‐6‐prop‐1‐en‐2‐ylbenzo[a]phenazine	0	−8.1	n.f.	—	—
NuBBE_1733	(1Z,4E)‐Germacrene B	1	−8.0	50.5	287.2	7.344
NuBBE_2273	(E)‐*d*ihydrooccidentalol	1	−8.0	61.1	336.0	0.3473
NuBBE_1352	gamma‐Eudesmol	1	−7.9	62.8	301.1	0.5199
NuBBE_1404	delta‐Elemene	0	−7.9	47.6	258.2	22.173
NuBBE_1542	Bicyclogermacrene	1	−7.9	48.5	267.8	15.56
NuBBE_1922	β‐Selinene	1	−7.9	48.1	262.9	18.52
NuBBE_2502^a^	C=C1CC[C@@H]2 C(=C)C(=O)O[C@H]2[C@@H]2 C(=C)CC[C@@H]12	0	−7.9	63.2	383.7	0.09106

^a^ SMILES notation due long name.

It is important to state that 16 molecules of 20 highlighted were classified as attractants in the ML model (Table 4). These molecules represent diverse structural classes and physicochemical profiles and are now cataloged as high‐priority candidates for future in vivo evaluation and behavioral assays.

A notable strength of this study lies in the convergence between the in silico screening results and some previously reported biological data, even if indirectly. α‐copaene and β‐chamigrene, sesquiterpenes identified as affinity ligands for *C. capitata* OBPs and classified as attractant have already been associated directly or indirectly in the literature with behavioral responses in fruit flies or other Diptera insects (Kharrat et al. 2025; Guimarães et al. 2025). This agreement helps to trust the generalization ability of the applied in silico methods. However, the complexity in protein recognition of individual or blends, in a diffuse manner, including different isoforms of receptors, makes ligand‐based methods such as ML‐QSAR more suitable for even more fruitful generalizations, by scanning the phase space for intrinsic characteristics of compounds that have proven to be good attractants, and are less subject to the imprecision inherent in docking methods.

## 4 Conclusion

This study integrated modern in silico techniques to identify and prioritize potential semiochemicals with attractant properties for *Ceratitis capitata*. By combining molecular descriptor generation with feature selection using the Bee Colony Optimization algorithm followed by Best‐First Search (BFS), a reduced subset of five relevant descriptors was identified. These variables effectively captured physicochemical and topological characteristics associated with molecular ability in olfactory mechanisms. The subsequent development and validation of an XGBoost‐based QSAR classification model enabled accurate prediction of attractant activity, achieving robust performance metrics, including high AUC scores in cross‐validation. The model predicted 206 natural compounds from the NuBBE database as more potential attractants, and a downloadable spreadsheet with prediction probabilities is provided to support future experimental validation.

The integration of ligand‐based descriptors, machine learning, and curated chemical databases demonstrated an efficient strategy for pre‐screening bioactive candidates in the context of olfactory protein targets. This workflow can significantly reduce the time and resources required for in vivo bioassays.

Molecular docking calculations brought information about the structural and energetic characterization of compounds in the odorant protein. The hydrophobic profile of the standard represented by farnesene was evidenced mainly in molecular dynamics simulations, reflecting a maintenance of the ligand in a region similar to the starting geometry from docking, denoting stability. An interaction energy value greater than 130 kJ/mol (in absolute magnitude) can be attributed to the enhancement of the non‐polar contributions in the MM/PBSA calculation, due to the characteristics of the ligand and OBP. Virtual screening via docking revealed 20 molecules with high attractant potential by the affinity criterion, satisfactorily confirmed by the Machine Learning model, with the exception of four that were classified as non‐attractants.

Overall, this study contributes to the field of chemical ecology and pest management by providing a reproducible cheminformatic framework and a curated library of potential attractants for *C. capitata*, supporting the development of sustainable monitoring and control strategies.

## Author Contribution

All authors contributed to the study. Conceptualization and implementations was made by Edilson B.A. Filho, Rosalvo F. Oliveira Neto, Beatriz A.G. Paranhos, Nathaly C. Aquino, and Ruth R. Nascimento. Material preparation, data collection, and analysis were performed by Ramiro G. Passos, Edilson B. Alencar Filho, Vanessa C. Santos, and Ana B.P. Bispo. The first draft of the manuscript was written by Edilson B. Alencar Filho, and all authors commented on previous versions of the manuscript. All authors read and approved the final manuscript.

## Conflicts of Interest

The authors declare no conflicts of interest.

## Supporting information

Supplementary_Information_AIBP_AlencarFilho2025.
